# Identification of a variant form of tyrosine phosphatase LYP

**DOI:** 10.1186/1471-2199-11-78

**Published:** 2010-11-02

**Authors:** Shaofeng Wang, Hongbo Dong, Jiayu Han, Wanting T Ho, Xueqi Fu, Zhizhuang J Zhao

**Affiliations:** 1 Department of Pathology, University of Oklahoma Health Sciences Center, Oklahoma City, Oklahoma 73104, USA; 2 Edmond H. Fischer Signal Transduction Laboratory, College of Life Sciences; 3 School of Pharmaceutical Sciences, Jilin University, Changchun, PR China

## Abstract

**Background:**

Protein tyrosine phosphatases (PTPs) are important cell signaling regulators with major pathological implications. LYP (also known as PTPN22) is an intracellular enzyme initially found to be predominately expressed in lymphocytes. Importantly, an allelic R620W variant of LYP is strongly associated with multiple autoimmune diseases, including systemic lupus erythematosus, rheumatoid arthritis, type 1 diabetes, and autoimmune thyroid disease.

**Results:**

In this study, we isolated a novel isoform of LYP designated LYP3. LYP3 differs from LYP1, the known isoform of LYP, in that it lacks a 28 amino acid segment right after the R620W site embedded in a proline-rich protein-protein interaction motif. Genomic sequence analysis revealed that LYP3 resulted from alternative splicing of the LYP gene located on chromosome 1p 13.3-13.1. Reverse transcription PCR analyses of 48 human tissues demonstrated that both LYP1 and LYP3 are predominantly expressed in primary and secondary lymphoid tissues but the relative expression levels of the two isoforms varies in different human tissues and individuals.

**Conclusions:**

We thus identified a new variant form of LYP and conducted a comprehensive analysis of LYP tissue expressions. Considering the pathogenesis of LYP R620W, we believe that the expression of LYP3 may have an important role in regulating activity and function of LYP and may be implicated in autoimmune diseases.

## Background

Protein tyrosine phosphatases (PTPs) act in a coordinated manner with protein tyrosine kinases to control cell signaling thereby regulating various physiological processes [1]. Malfunctioning of these enzymes has major pathological implications. One of the best known examples is the allelic variant of the lymphoid tyrosine phosphatase LYP (PTPN22) which is associated with multiple autoimmune diseases, including systemic lupus erythematosus, rheumatoid arthritis, type 1 diabetes, and autoimmune thyroid disease [2,3].

LYP is a cytoplasmic enzyme belonging to the PEST group of non-receptor classical PTPs [4]. It contains 807 amino acid residues. The murine ortholog of LYP is called PEP [5]. LYP and PEP share 89% and 61% sequence identity in their PTP domains and noncatalytic portions, respectively. The N-terminal part of LYP/PEP contains the catalytic domain conserved in all classical PTPs. The structure of the sequence following the catalytic domain is largely undefined. The last 200 amino acid segment contains 4 proline-rich sequence motifs (P1-P4) which presumably provides docking sites for SH3 domain-containing signaling proteins. The first of these motifs, P1, is known to bind with relatively high stoichiometry to the SH3 domain of the Csk tyrosine kinase, an important negative regulator of T-cell antigen receptor signaling [6-8]. LYP/PEP and Csk appear to have complementary functions. While Csk phosphorylates the negative regulatory tyrosine residue at the C-terminal end of Lck and Fyn, LYP/PEP dephosphorylates the positive regulatory site in the middle of these two tyrosine kinases [9]. Both actions result in inhibition of TCR signaling. LYP/PEP also negatively regulates T cell signaling by dephosphorylating Zap-70 and the tyrosyl residues within the immunoreceptor tyrosine-based activation motifs in CD3 ζ-chains, which are phosphorylated by Src family tyrosine kinases [10]. Further studies by using a substrate-trapping mutant of LYP in combination with mass spectrometry identified the following substrates: Lck (at Y394), ZAP-70 (at Y493), Vav, valosin-containing protein, and immunoreceptor tyrosine-based activation motifs in CD3 ζ-chains [11]. LYP/PEP is predominately expressed in lymphocytes, and it is generally defined as a key inhibitor of T-cell activation.

A major advancement in the study of LYP is the finding of a single nucleotide polymorphism (SNP), namely, C1858T, which generates an R620W amino acid substitution (reviewed in 2, 3). This SNP is a common risk factor for many autoimmune diseases, including type I diabetes [12], systemic lupus erythematosus [13], rheumatoid arthritis [14,15], and Graves' disease [16]. The 620 amino acid residue is located within the first protein-rich motif P1, which interacts with the SH3 domain of Csk [6-8]. The R620W amino acid substitution presumably interrupts the interaction of P1 with the SH3 domain of Csk [12]. However, a later study revealed that the R620W substitution is actually a gain-of-function mutation that generates a more active PTP that acts as a more effective inhibitor of T-cell signaling than the wild-type enzyme [17]. The mechanism by which this R620W substitution in the non-catalytic segment leads to activation of the phosphatase remains to be defined. It was postulated that this activating mutation in LYP may cause a predisposition to autoimmune disease either by failure to delete auto-reactive T cells or due to insufficient activity of regulatory T cells [17]. In any event, the C-terminal part of LYP has a crucial role in regulating the catalytic activity of LYP and its physiological functions. In this study, we isolated a new variant form of LYP designated LYP3 which differs from LYP1, the known isoform of LYP, in the C-terminal region right after the R620W substitution site. We found that the expression level of LYP3 relative to LYP1 varies in different tissues and among different individuals.

## Methods

### Materials

Total RNAs were isolated from de-identified human peripheral blood samples by using the Trizol reagent (Invitrogen) and were then treated with RQ1 RNase-free DNase (Promega) to remove contaminated genomic DNAs. First-strand cDNA was synthesized by using the iScript cDNA Synthesis Kit (Bio-Rad) with random primers. Human Major Tissue qPCR Array containing first strand DNAs from 48 tissues was purchased from OriGene.

### Molecular cloning of LYP

Based on the coding sequence of LYP in GenBank database, two PCR primers, namely, Lyp5' (5'-GACATGCCCTCCCTCAACCTACTTA) and Lyp3' (5'-TGCAGGTGTACTTGCAGCCCATATTA) were synthesized. They correspond to the 5' and 3' ends of the coding sequence of LYP1, respectively. Single-strand cDNAs synthesized with total RNAs purified from human peripheral blood samples were used as templates. The PCR was run for 35 cycles with Phusion polymerase (Finnzymes) at 94°C for 30 sec, 65°C for 30 sec, and 72°C for 2 min. The PCR products were ligated with T4 DNA ligase into the pBluescript KS vector which was opened by digestion with EcoRV. Clones were selected based on blue-white selection, and plasmid DNAs were isolated for sequencing analyses with T3 and T7 primers by using the automatic DNA sequencing facility at the Oklahoma Medical Research Foundation.

### Determination of tissue distributions of LYP1, LYP2, and LYP3

To determine the expression of LYP1, LYP2, and LYP3 in various human tissues, we employed the Human Major Tissue qPCR Array (OriGene) containing first strand DNAs from 48 tissues. The PCR primers were L1 (5'-CTAGCAACTGCTCCAAGGATAGATGA), L1r (5'-GCTTGTTTGGTGGGCAAGAATTACA), L2r (5'-GGGCTAAATGTCATCTAAAGCCAAG), and L2 (5'- TCCTCAGCTGTGAAGAGTGTAAAACTC). These primers were derived from different exons on the LYP gene and are expected to give rise to distinct PCR products for LYP1, LYP2, and LYP3 cDNAs. L1 and L1r produce 505 and 421 bp fragments from LYP1 and LYP3, respectively; L1 and L2r amplify a 284 bp fragment from LYP2 only; L2 and L1r specifically yield a 301 bp fragment of LYP3. The PCR was run for 35-45 cycles, and the conditions were 94°C for 20 sec, 63°C for 20 sec, and 72°C for 1 min. The products were analyzed on 1.5% agarose gels stained with ethidium bromide or on 8% acrylamide gels visualized by using silver staining.

## Results and Discussion

### Identification of LYP3, a variant form of LYP

**Figure 1:**
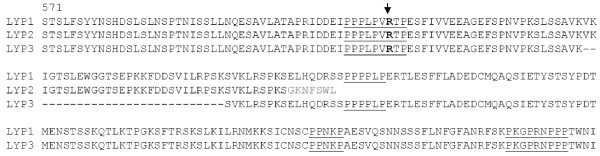
**Sequence alignment of three isoforms of LYP at the C-terminal region starting from amino acid residue No. 571**. The four proline-rich motifs are underlined. The pathogenic R620W substitution is shown in bold and indicated by an arrow.

In order to clone the full-length form of LYP1, we designed a pair of primers that cover the entire coding sequence of LYP1 (GenBank accession no. NM_015967). Single-strand cDNAs from a human peripheral blood sample were used as templates for PCR, and the PCR products were cloned into the pBluescript KS vector. Interestingly, DNA sequencing analyses revealed clones carrying the expected LYP1 but also a variant form of LYP which is 84 bp shorter in cDNA corresponding to a 28 amino acid deletion. Since there is a variant form of LYP2 in the GenBank database (NM_012411), we designate the new isoform LYP3. The DNA sequence of LYP3 has been deposited in the GenBank database under accession no. GU479452. Figure 1 shows a sequence alignment of amino acid residues of the three forms of LYP at the C-terminal regions where these three isoforms differ. LYP1 contains an N-terminal PTP domain, a central region of unknown function, and a C-terminal portion of approximately 200 amino acids containing four proline-rich motifs termed P1-P4. P4 is part of the so-called C-terminal homology (CTH) domain, which is found in all members of the PEST group of PTPs [1,2]. LYP3 differs from LYP1 in that it is missing 28 amino acid residues in between the P1 and P2 region. P1 is known to interact with the SH3 domain of the Csk tyrosine kinase, an important negative regulator of T-cell antigen receptor signaling [6-8]. Shortening of the P1-P2 linker region may affect the CSK/LYP interaction and other unidentified interactions. It should be noted that the C1858T SNP happens to cause the R620W substitution in the P1 region. In addition, LYP2, another variant form of LYP, has an even shorter C terminus lacking P2, P3, and P4.

### LYP3 is an alternative splicing product

**Figure 2:**
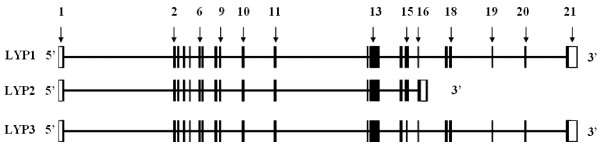
**Schematic diagram of the gene structure of three isoforms of LYP**. Exons are represented by bars and drawn approximately proportional to their sizes and positions. The open part of bars stands for non-translational regions. The numbers of exons are selectively labeled based on the LYP1 coding sequence. Note that LYP3 has a short exon 15 and LYP2 contains a longer exon 16 with an in-frame stop codon. All the intron-exon junctions follow the GT-AG rule.

**Figure 3:**
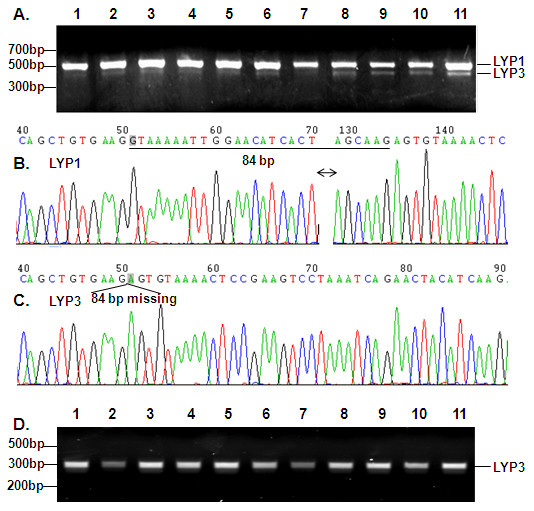
**PCR amplification of LYP1 and LYP3 and verification of PCR products by DNA sequencing**. A. Single-strand cDNAs obtained from peripheral blood of 11 individuals were amplified with primers L1and L1r which give rise to both LYP1 and LYP3. PCR products were resolved on 1.5% agarose gels and were detected by staining with ethidium bromide. B. and C. LYP1 and LYP3 PCR products shown in panel A above were purified by using 8% polyacrylamide gel and re-amplified with primers L1 and L1r for sequencing analyses with primer L1. Note that underlined sequence of LYP1 in panel B denotes the 84 bp segment missing in LYP3. The sequencing data represent all LYP1 and LYP3 bands shown in Fig. 3A and Fig. 4. D. LYP3 was amplified from the 11 blood cDNA samples shown above in panel A with primers L2 and L1r. PCR products were resolved on 1.5% agarose gels and were detected by staining with ethidium bromide.

A search of the human genome database by using the BLAST program revealed that LYP3 is a result of alternative RNA splicing. Both LYP1 and LYP3 consist of 21 exons with the translation initiation and termination codons residing in the first and last exons, respectively. All the intron-exon junctions follow the GT-AG rule. LYP1 and LYP3 differ in exon 15 where LYP3 is 84 bp shorter. Figure 2 also shows the exon-intron structure of LYP2. LYP2 has a longer exon 16 which contains an in-frame termination codon. To verify the presence of LYP3, we designed primers L1 and L1r that amplify both LYP1 and LYP3. PCR results demonstrated the presence of the shorter LYP3 product in blood samples (Figure 3A). Interestingly, the relative ratios of LYP1 and LYP3 appeared to differ among samples from different individuals. DNA sequencing analyses of gel-purified PCR products confirmed that the shorter product indeed corresponded to LYP3 which is 84 bp shorter than the longer LYP1 PCR product (Figure 3B,C). We also employed a pair of primers (L2 and L1r) to amplify LYP3 specifically. Data in Figure 3D illustrated a single band of expected size for all 11 blood samples. These blood samples analyzed here were collected from various de-identified normal volunteers and patients. We do not yet know if these differential expressions of LYP1 and LYP3 correlate with any disease phenotype.

### Distribution of three isoforms of LYP in human tissues

**Figure 4:**
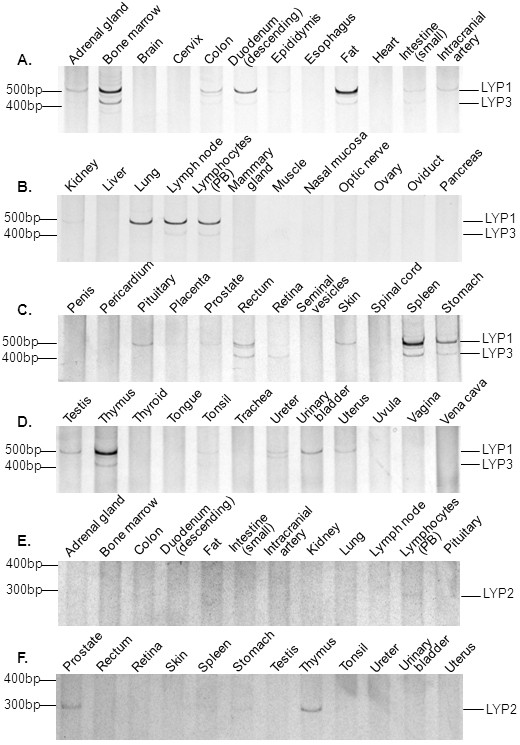
**Distribution of three isoforms of LYP in 48 human tissues**. First-strand cDNAs in the Human Major Tissue qPCR Array kit (OriGene) were amplified by PCR with primers L1/L1r for LYP1 and LYP3 and L1/L2r for LYP2 as described in Materials and Methods. PCR products were resolved on 8% polyacrylamide gel and were detected with silver staining. The expected LYP1, LYP2, and LYP3 PCR products are indicated. For simplicity, only selected data were shown for analysis of LYP2.

As its name indicates, LYP was initially thought to be expressed specifically in lymphocytes. This was based on the Northern blotting analyses of a limited number of human tissues (spleen, thymus, prostate, ovary, testis, small intestine, and colon) and cell lines [4]. However, subsequent studies revealed expression of LYP in normal granulocytes and monocytes and a number of myeloid cell lines [18]. We felt that it was necessary to conduct a more comprehensive analysis of LYP expression. For this purpose, we employed the human Major Tissue qPCR Array from OriGene. The array contains the first strand cDNAs of 48 human tissues. The cDNAs have been normalized with house keeping genes, and thus PCR data represent relative expressions of specific genes in different human tissues. We first performed PCR with primers that cover both LYP1 and LYP3. The data shown in the upper four panels of Figure 4 demonstrate that both LYP1 and LYP3 are highly expressed in major lymphoid tissues (bone marrow, lymph node, peripheral blood, spleen, and thymus). They are also expressed at a high level in lung and fat and at lower levels in many other tissues including adrenal gland, colon, duodenum, intestine, intracranial artery, pituitary, rectum, skin, stomach, testis, tonsil, ureter, urinary bladder, and uterus. These tissues can be considered as the mucosa-associated lymphoid system which contains small concentrations of diffuse lymphoid cells. However, it is hard to attribute all the expression of the LYP isoforms to the presence of lymphocytes in lung and fat considering the level of expression. Further immunohistochemical study is required to clarify the expression LYP1 and LYP3 in non-lymphocytes. Nonetheless, it is clear from our data that LYP1 and LYP3 are not expressed in major non-lymphoid tissues such as brain, heart, liver, and muscle. LYP1 appears to be the major form in most of the lymphoid tissues, and the ratios between LYP1 and LYP3 vary in different tissues. Interestingly, however, the retina only expresses LYP3. It is also worth noting that LYP3 is not present in the lung. Overall, our data generally support the notion that LYP1 and LYP3 are expressed in primary and secondary lymphoid tissues. We also synthesized PCR primers to amplify specifically the reported LYP2 isoform. LYP2 was initially cloned from a human thymocyte library [4]. It contains a shorter C terminus due to the presence of an earlier termination codon in exon 16 (see Figure 1 and 2). However, no further follow up work was done. Our data indicate that LYP2 indeed exists, but only at very low levels in peripheral blood lymphocytes, thymus, and interestingly in prostate (Figure 4, two lower panels).

DNA sequence variations play a fundamental role in human phenotypic variability, including susceptibility to diseases. SNPs represent the most extensively studied sequence variations. Non-synonymous SNPs change the amino acid sequence and presumably alter protein functions. The C1858T SNP that leads to the R620W substitution in LYP is a typical example. On the other hand, alternative splicing of mRNA may generate protein isoforms of different sizes with different biological properties such as protein/protein interaction, subcellular localization, and catalytic activity [19,20]. We believe that this should be the case for LYP2 and LYP3. To ensure correct cellular functions, gene expression is finely regulated both spatially and temporally, and the importance of aberrant RNA processing in diseases has been generally recognized. Pre-mRNA splicing is a tightly regulated process affected by many factors including SNPs [21]. In this regard, it should not be a surprise if the C1858T SNP affects expression of alternative spliced forms of LYP. The C1858T SNP is present mainly in Caucasian populations and is essentially absent in African and Asian populations [2]. It is not known if other DNA variations in LYP affect autoimmune diseases in the latter populations which have no fewer incidences of these diseases. Our data indicate that the relative ratios of LYP isoforms vary among different individuals. It will be important to find if aberrant expression of LYP isoforms cause any human diseases, particularly autoimmune diseases.

## Conclusions

To conclude, extensive literatures exist to support the association between LYP and multiple autoimmune diseases. However, the underlying molecular mechanism is still elusive. We have now identified a novel isoform of LYP designated as LYP3. This allows us to look at the problem from a new angle.

## Competing interests

The authors declare that they have no competing interests.

## Authors' contributions

SW, HD, JH, and WTH conducted the research experiments; XF designed the experiments; ZJZ designed the experiments and wrote the manuscript. All authors read and approved the final manuscript
